# End-of-Life Care Training for Patients with Traumatic Brain Injury in Ghana: A Novel Curriculum and Its Initial Implementation

**DOI:** 10.3390/jcm14113643

**Published:** 2025-05-22

**Authors:** John Bruno, Mayur Patel, Rebecca Henderson, Michael Mathelier, Taylor N. Smith, Joseph C. Pompa, Cassandra Clay, Marie-Carmelle Elie, Sheba Afi Mansa Fiadzomor, Lawrence Nsohlebna Nsoh, Torben K. Becker

**Affiliations:** 1 Division of Critical Care Medicine, Department of Emergency Medicine, College of Medicine, University of Florida, 1600 Archer Road, Gainesville, FL 32608, USA; 2 Section of Global Health, Department of Emergency Medicine, College of Medicine, University of Florida, 1600 Archer Road, Gainesville, FL 32608, USA; 3 Department of Neurology, University of Florida, 1600 Archer Road, Gainesville, FL 32608, USA; rrhenderson@ufl.edu; 4 Department of Obstetrics & Gynecology, University of Alabama, Tuscaloosa, AL 35487, USA; 5 College of Medicine, University of Florida, 1600 Archer Road, Gainesville, FL 32608, USA; 6 College of Nursing, University of Florida, 1600 Archer Road, Gainesville, FL 32608, USA; 7 Department of Emergency Medicine, Saint Francis Hospital, Roslyn, NY 11576, USA; 8 Department of Emergency Medicine, University of Alabama at Birmingham, Birmingham, AL 35294, USA; dr.s.fiadzomor@gmail.com (S.A.M.F.); nsohabolga@gmail.com (L.N.N.); 9 Ghana Armed Forces Medical Services, Ghana

**Keywords:** palliative care, low- and middle-income countries, end-of-life, traumatic brain injury

## Abstract

The implementation and practice of palliative medicine have numerous boundaries in low- and middle-income countries (LMICs), stemming from various cultural, legal, and religious concerns. Additionally, professional education in palliative care medicine in these countries is severely lacking, especially when compared with developed countries. **Background/Objectives**: To enhance and demystify palliative medicine practice to health care providers in LMICs. **Methods**: We developed a novel and comprehensive course in palliative care medicine and end-of-life (EOL) care, specifically within the context of management of patients with traumatic brain injury (TBI). We performed both immediate pre-course and post-course analysis of course participant comprehension and feedback, as well as a one-year post-course analysis and small group discussion. **Results**: The comprehension of the course material was strong, as participants scored an average of 13.9 points better on the post-test compared to the pre-test (49.6% vs. 35.7%, *p* < 0.001). Participants in the one-year follow-up session reported long-term applicability of the course material in their respective practice settings, with all participants reporting that they utilize the course material often. Small group discussion responses indicated a strong level of comprehension of the course material. **Conclusions**: Providing education in palliative medicine to health care professionals in LMICs is feasible, and likely to be both well-received and strongly influential to local medical practice. Local cultural and religious practices may be less of a barrier to the provision of palliative medicine than previously considered. Practicing palliative medicine, particularly at EOL, may strengthen patient–provider relationships, improve job satisfaction among health care providers, and improve the perception of medical care provided in LMIC medical settings.

## 1. Introduction

Morbidity and mortality are common after hospital admissions worldwide, especially considering an aging global population and the increasing prevalence of chronic end-stage conditions requiring high levels of care. This is especially true in low- and middle-income countries (LMICs), in particular sub-Saharan African countries, where in-hospital mortality can be as high as 40-fold than that seen in high-resource countries, owing to a high burden of severe disease in combination with limited availability of medical resources [1]. The provision of palliative care in conjunction with curative care has been demonstrated to reduce unnecessary treatments or procedures, reduce health care costs, and improve patient satisfaction without increasing overall mortality [2,3,4]. Evidence dating back more than 40 years supports that the provision of palliative care is beneficial not only to those with end-stage and life-limiting diseases as referenced above, but also to patients with other serious illnesses or injuries, both acute and chronic [5].

Most of the available literature on palliative medicine comes from North America, Europe, and Australia, with a vast majority of publications emerging from the United States. An urgent need for expanded access to palliative care has been identified in LMICs [5]. Similarly, while the practice of palliative medicine has seen significant advances in Western medicine, its utilization in LMIC medical practice has lagged behind substantially. The World Health Organization (WHO), which has identified palliative care as a basic human right, identified in 2011 over 34 million people who died from diseases requiring palliative medicine. This number is likely underestimated considering the breadth of diseases that may benefit from palliative care, as well as the underreporting of epidemiologic statistics [5,6]. Current estimates indicate that as high as 80% of the world’s palliative care needs come from LMICs, a number that may increase further as the world’s population ages [7]. However, its implementation, and consequently patient access to this highly beneficial care, are often highly limited. This is owing to local legal, cultural, and religious factors in addition to the paucity of literature arising from these regions [6,7,8,9,10,11,12]. Resource limitations also drive significant disparities regarding the availability of clinicians with dedicated end-of-life skills and knowledge, access to appropriate pain management solutions, and a lack of public policies and national health system support for patients at the end of their lives. Furthermore, formal or even informal education in palliative medicine is either lacking or non-existent in many LIMC teaching facilities [13,14,15,16]. Similarly, health care professionals in LMICs frequently view their knowledge and training in palliative concepts as notably poor [17,18,19,20].

The burden of critical illness due to trauma, including those with traumatic brain injury (TBI), is high in LMICs [21,22]. Ghana, a West African country with a population of approximately 31 million, is a stable democracy and is classified as a low- to middle-income country (LMIC). Many of the challenges in end-of-life care mentioned above limit patients’ access to palliative medicine in Ghana. The Ghana Armed Forces Medical Services provide medical and surgical care, including emergency and intensive care unit services, to members of the military and civilians at military hospitals throughout the country and in other countries as part of peacekeeping and related operations [23]. The incidence of TBI is high in Ghana, due to a high burden of motor vehicle crashes (MVCs) [21]. Given the high burden of palliative care needs in patients suffering from TBI, we identified this patient population as of particular interest and of high potential for successful utilization of palliative care practice and techniques in Ghana [24]. Next, we describe the implementation of a short, comprehensive training course in the principles and practice of palliative and end-of-life care for patients with TBI, presented to health care professionals of multiple disciplines employed at a single urban military medical center in Ghana. Finally, we report on both immediate and one-year feedback and impressions from the students who attended the program.

## 2. Methods

We developed the end-of-life (EOL) care in the TBI didactic course as a curriculum containing 12 lectures, interspersed with 4 small group sessions designed to reinforce the lecture material (Table 1). Lectures were written by the authors based on current literature and best practice guidelines. The material was adapted as best as possible toward practice in a low-resource setting, with the omission of some diagnostic and treatment modalities unavailable or not easily available in LMICs. The lectures were presented by primarily emergency medicine physicians, most formal subspecialty training in either global emergency medicine, emergency medical services, emergency ultrasound, hospice and palliative care, critical care medicine, and neurology critical care medicine, with several authors having formally trained in several of the above subspecialties. The didactic material was presented over two days of lecture, loosely divided so that day 1 was focused on principles of TBI and issues related to complications and prognosis, and day 2 was focused on palliative medicine and end-of-life related issues (Table 1). TBI lecture topics specifically were written with a particular focus on the identification and control of distressing symptoms. The third and final day of the course was dedicated to group sessions and simulation, designed to incorporate all of the palliative care principles learned in the didactic portion of the course, with a focus on EOL care communication topics such as breaking bad news, goals of care discussions, and difficult family interactions.

A pre-test was administered, and an identical exam was given at the course’s conclusion to assess the comprehension of the didactic materials. Similarly, a pre- and post-course survey was administered to assess satisfaction with and potential applicability of the course material. Finally, a one-year follow-up small group discussion and survey were held with the course director and volunteers from the pool of students who had taken the course, during which another survey was administered (Appendix D). In this session with a focus group format, course graduates discussed if and how the course had affected their practice. They were also given the opportunity to discuss specific cases or scenarios in which the course material was utilized.

## 3. Results

A total of 33 health care professionals took the EOL care course as a multidisciplinary course consisting mostly of physicians of multiple levels of training, including attending physicians, clinical psychologists, and nurses. A total of 32 participants’ data were available for the pre-test and pre-course survey, and data were available for 31 participants for the post-test and post-survey (Appendix A, Appendix B and Appendix C). One post-test was omitted from the final analysis because the identity of the test-taker could not be determined, and thus it could not be correlated with a corresponding pre-test. Exams were scored with a total of 100 points achievable. Participants scored an average of 13.9 points better on the post-test compared to the pre-test (49.6% vs. 35.7%, *p* < 0.001; Table 2).

In the pre-survey, participants selected their pre-course knowledge, comfort level, and exposure to palliative care practices on a scale of 1–5. On average, participants ranked their pre-course palliative care knowledge as 2.5/5 (SD 0.9), indicating between “below average” and “average” knowledge. They indicated the frequency at which they practiced palliative-related medicine between “once per month” and “once per week”. Average pre and post-test scores are available in Table 3.

In the post-survey, participants assessed the content of the course (Appendix B). Notably, confidence in the course content was assessed on a scale of 1–4, with 4 being the highest. Course participants rated their post-course knowledge of the TBI content as an average of 3.7 (SD 0.5), and their knowledge of the palliative care content as an average of 3.8 (SD 0.4). Participants ranked the effectiveness of the lecture material and the small group sessions, on a scale of 1–5 (with 5 being the highest), an average of 4.6 (SD 0.8) and 4.7 (SD 0.8), respectively. Despite the intentional difficulty of the course material, pre-test and post-test, participants ranked the difficulty of the course as an average of 3.0 (SD 0.3), indicating a desired amount of difficulty, with a score of 1/5 indicating a course that was too difficult, and 5/5 indicating material that was too simple. Please see Appendix B.

A total of eleven participants volunteered to participate in the one-year follow-up session (Table A1 in Appendix E). In the one-year survey, participants reported on average that their knowledge base regarding TBI had greatly improved since taking the course (4.63/5), but reported variability regarding how often this knowledge was utilized (3.22/5). Participants noted that many of them are infrequently in settings in which they can manage TBI patients, except for participants who work in the emergency department.

In contrast, participants noted that they utilize the course material on palliative care and end-of-life communication highly frequently, with all participants noting that their perception of palliative medicine has greatly changed (5/5) since taking the course. All participants noted that they use content and strategies learned from the course in breaking bad news often (5/5). Participants reported that they utilize concepts learned from the palliative medicine section of the course highly frequently (4.9/5), with 9/11 participants noting that they use concepts learned in this course daily, including each of the nurses interviewed (Table 4). However, only 3/11 participants in these discussions reported using shared decision-making principles as taught in the course. Additionally, important qualitative data were also collected during this encounter, and will be discussed below.

## 4. Discussion

The World Health Organization has declared access to palliative medicine as a human right [5,7,25]. It defines palliative care as “… A care approach that improves the quality of life of patients and their families who are facing problems associated with a life-threatening illness, through prevention and relief of suffering by means of early identification and impeccable assessment of pain and other problems, physical, psychosocial and spiritual” [25,26]. Importantly, early provision of palliative care, particularly in the setting of critical illness, does not negatively impact overall mortality [2,3]. Despite this, the delivery of palliative care education to health care professionals working in LMICs, and consequently improving local patient access to palliative services, have been challenging [5,6,7,8,13,19,27].

We present the results of the implementation of a three-day lecture series focused on palliative care medicine to a class of health care professionals employed at a large military hospital in Ghana. Specifically, the course material was prepared in the context of traumatic brain injury patients—a patient population that is very common in Ghana. While there is literature describing the practice of palliative medicine, formal training in palliative medicine is lacking in Ghana. Our course thus provided a novel educational experience to participating Ghanaian health care providers in EOL medicine, which has not been previously described in the literature [11,12,28,29]. While there is formalized palliative education in some LMICs, for instance, South Africa, to our knowledge, this is the first documented literature on the implementation of such a curriculum to students in an LMIC setting without previous training in palliative management [14,27]. Notably, the students’ reflections on the content both immediately after the course and at a one-year follow-up after having the opportunity to potentially practice the knowledge learned provide unique insights into the impact of practice that even such a short course can provide.

Response to the course was overwhelmingly positive among participants, as evidenced by the improvement in test scores after taking the course, the post-test survey, and most importantly in the one-year follow-up. Immediate post-course test and survey results suggest both an appreciation of the course material presented, as well as a perceived relevance toward their practice settings. Perhaps more importantly, the one-year follow-up survey and interview results suggested a deep and foundational understanding of the course material presented, as evidenced by the high levels of practical application reported by course participants, and their perception of resultant improvements in their day-to-day practice.

The positive response was most evident in the context of material relating to palliative medicine, breaking bad news, and end-of-life care. Participants, who initially reported utilizing palliative care practice between once monthly and once weekly in the pre-test survey, highly utilized the concepts after the course completion. All participants interviewed at the one-year follow-up reported using a model, such as the SPIKES model (Setting-Perception-Invitation-Knowledge-Emotion-Summarize) discussed in the course, to aid in breaking bad news [30]. Participants noted that while they had previously considered breaking bad news to be one of the worst and most frustrating parts of their job, they now approach such situations and difficult conversations favorably as they view themselves as having a certain level of expertise in the subject. Participants also perceived that utilizing the palliative care approach from the course seemed to improve family and patient satisfaction. This is consistent with previous literature that suggests incorporating palliative medicine into routine practice is associated with increased job satisfaction in health care professionals [6,20]. Course participants also note that families seem to respond more positively than expected to bad news when presented using the tools and strategies discussed in the course. Interestingly, participants also noted that when coworkers who did not take the course are tasked with breaking bad news to families or having goals of care conversations, participants are now more likely to notice flaws in these approaches, and more importantly, consequent negative effects on and reactions from the family members involved in these discussions.

Interestingly, the material regarding shared decision-making was not as well received as the other course material and was not utilized with any notable frequency, according to participants in the one-year follow-up. Participants pointed to several factors that influenced this pattern. Firstly, they note that families often request therapies that are not evidence-based, including home remedies, or therapies that are either unavailable or unlikely to benefit the patient. They report that family members commonly have negative reactions when these therapies are not offered after attempts at shared and evidence-based informed decision-making. This is consistent with the experiences of health care professionals at other medical centers in LMICs [6,31]. Secondly, participants note that the up-front fee-for-service payment model at Ghanaian hospitals may influence families to choose less expensive treatment modalities, even though these specific modalities may not provide a benefit or may even be harmful to patients at the end of life. For instance, a participant noted that when families ultimately decide to pursue withdrawal of life-sustaining therapy, if given a choice, family members may elect not to treat the patients’ pain at the end of life due to financial constraints. Notably, narcotic analgesia medication remains expensive and less available in LMICs [32]. It is possible that as palliative medicine provision evolves in LMICs, and access to palliative care medications improves, shared decision-making may develop value in patient care. However, the role of shared decision-making would seem to have several important factors limiting its utility.

Initially, participants expressed skepticism during the course’s small group discussions that local cultural and religious practices may limit the ability to apply communication principles primarily derived in high-resource settings, specifically regarding topics involving death and morbidity. Indeed, local cultural and religious concerns have been previously cited as potential barriers to practicing palliative medicine [7,10,20,33]. However, these concerns may be less important than previously considered. Based on our findings, except for the content on shared decision-making, these considerations appear not to have significantly factored into the participants’ ability to impact patient care using palliative care principles. Participants noted that the palliative approach to breaking bad news and end-of-life care frequently triggered positive involvement in family religious and cultural practice, often helping family members come to terms with a bad outcome, and finding solace in their religion as a foundation to cope. Based on our findings, breaking bad news in a regimented fashion, and utilizing a palliative care approach toward end-of-life issues as taught in this course may ultimately help to positively engage the cultural and religious beliefs and practices of families. However, this area of inquiry requires more research before attempting to generalize these results, especially in regard to the legality of such practice locally, and the potential reactions of local communities and government bodies to the practice of palliative medicine [20,33].

### Limitations

There are several limitations to this study. The primary intent of this project was to report on the feasibility of a dedicated palliative care course, and specifically its effect on the practice patterns of Ghanaian health care providers. We did not intend this to be a clinical study, and for this reason, we did not investigate patient-centered outcomes such as effects of our course on mortality, lifespan, health care costs, or patient satisfaction at EOL. These data would be highly useful, and further studies on the topic would be beneficial to understanding the long-term effects on patients from the implementation of palliative medicine practice at Ghanaian health care facilities. Previous data available suggest that these patient-centered outcomes would have a high likelihood of being improved as a consequence of the implementation of our course [2,3,34].

Regarding the course itself, the level of difficulty (oriented towards physicians) of content material, including pre-/post-test material, may have led to lower comprehension, and therefore less clinical utilization, of concepts by participants with less rigorous education, such as nurses. However, post-test scores still indicated comprehension in these individuals, and post-course survey data suggested that the course was at a desired level of complexity. Notably, feedback provided by non-physician participants in the one-year follow-up group discussion indicated high levels of comprehension of the source material, suggesting that this material was certainly suitable for presentation to a non-physician audience. The creation of courses specifically tailored to different health care professions may improve comprehension. However, we observed that a multidisciplinary course led to highly valuable and meaningful small-group discussions and simulation experiences and may have enhanced the reflection of the course material.

While Ghana is home to many different languages and dialects, many Ghanaians speak English, and course participants were proficient in speaking English. Language barriers have been identified as a barrier to the practice of palliative medicine, with notable concern that palliative care terms occasionally do not translate well into other languages, and these translations may sound scary or stoke fear in patients or family members [7]. It is possible that the presentation of course material, and as a consequence practical implementation, may have more limitations if participants and/or local community members are less proficient in English in other LMICs.

The study is also subject to several sources of selection bias. First, this course was implemented at a single center at an urban, military teaching hospital in Accra, Ghana. It is possible that this population of students, and downstream the population of patients to whom these participants provide health care, are not generalizable to other patient populations, specifically those seen in other LMIC settings. It is also plausible that in another LMIC region, local cultural, religious, and health care practices differ significantly enough that implementation in these regions would not have the same effect and may lead to encountering unexpected boundaries. Second, the course was taken voluntarily, which may have led participants with a pre-existing interest in palliative care principles to self-select this course.

More importantly, participation in the one-year follow-up was also voluntary. Of the 33 course participants, only 11 individuals attended the one-year follow-up, and their responses and feedback may not be representative of the views or practices of the entire group. Additionally, this one-year follow-up group discussion was facilitated by the course director himself, which introduces further potential bias, specifically in the survey results and prompt responses elicited from course participants. In addition to these potential biases, the total number of participants in the class was low. It is worth noting that participation in this course was voluntary, and course participants were initially skeptical of the course material. After receiving positive feedback from participants, we expect interest in this course material to be significantly higher for future class enrollment. Providing this course material or that of a similar course to a larger number of participants in a future study would be highly valuable to the available literature.

Finally, this course, while initially designed for physicians, was delivered to a multidisciplinary group of participants, which may not be reflective of the health care practitioners who are best suited to use this material and incorporate it into their practice. However, it should be noted that in other LMIC settings, physicians are not necessarily the health care professionals who are in charge of breaking bad news and initiating palliative-care-focused discussions as a default [17]. At the facility at which participants are employed, it is commonplace for either clinical psychologists, nurses, or non-physician staff such as midwives or dieticians, to be heavily involved with end-of-life discussions, and non-physician participants cite that they are frequently the ones to break bad news about patient conditions. Participants noted that oftentimes, nursing or clinical psychology personnel have more time to have these conversations with families and that physicians, particularly the physicians who did not take the course, are often either too busy to have dedicated goals of care conversations or do not recognize the importance of such conversations. They also note that, since taking the course, they have felt more empowered to discuss with consulting physicians the need to have goals of care discussions and initiate palliative care-focused treatment.

## 5. Conclusions

Implementation of a curriculum with the intent to provide education on palliative medicine in LMICs is feasible and can result in local adoption of the practice of palliative care principles similar to those used in high-income settings. Our study suggests that the practice of these learned principles may enhance the relationships between patients, families, and health care professionals and may improve career satisfaction, specifically regarding the care provided at the end of life by health care professionals. Local religious and cultural idiosyncrasies were perceived as less of a barrier to the implementation of palliative care education and practice than previously thought. The widespread adoption of palliative care principles at the end of life in LMICs would benefit from further study: it may reduce health care-related costs, and prevent unnecessary hospitalizations, all while improving quality of life, perception of care at the end of life, as well as patient–clinician relationships.

## Figures and Tables

**Table 1:** Didactic curriculum overview. Days 1 and 2 of the didactic curriculum. These days were subdivided into two half-day sessions, each comprising three lectures and a relevant small group session. The small group session format included a case, with prompts to facilitate interdisciplinary discussion. Each small group included a course educator in the role of facilitator. * Indicates a small group session title. ^1^ TBI: traumatic brain injury. ^2^ EOL: end of life.

Day 1	Day 2
Principles of TBI ^1^: Part 1	Introduction to Palliative Care: Part 1
Principles of TBI ^1^: Part 2	Introduction to Palliative Care: Part 2
Therapies for TBI ^1^	Brain Death: Definitions and Exam
* TBI management	* Ethical, Religious and Cultural Issues in EOL ^2^ Care
Paroxysmal Sympathetic Hyperactivity	Shared Decision-Making
Persistent Coma and Delirium	Breaking Bad News
Neuroprognostication	Medications at End of Life
* Effective communication with families regarding prognosis	* Withdrawal of Life-Sustaining Therapy

**Table 2:** Paired cohort sample size and pre/post average test scores (complete pre/post data).

Group	N (%)	Pre-Test Average Score	Post-Test Average Score	Paired T-Test *p*-Value
Physician	7 (26%)	44.0	58.9	
Nurse	11 (41%)	31.6	49.1	
Psychologist	3 (11%)	30.7	45.3	
Other *	5 (19%)	37.6	45.6	
Missing	1 (4%)	28.0	24.0	
TOTAL	27 (100%)	35.7/100 (SD 9.8)	49.6/100 (SD 13.3)	<0.001

* other consists of dietician, midwife, and physician-clinical psychologist. See Appendix C for the full content of the pre-/post-test.

**Table 3:** Total cohort sample size and pre/post average survey scores.

Group	Pre N (%)	Post N (%)	Pre-Survey Average Question Score **	Post-Survey Average Question Score **
Physician	10 (31%)	8 (27%)	3.1	4.1
Nurse	12 (38%)	13 (43%)	2.8	3.7
Psychiatrist	4 (13%)	4 (13%)	2.4	3.8
Other *	5 (16%)	5 (17%)	2.2	3.7
Missing	1 (3%)	0 (0%)	1.5	-
TOTAL	32 (100%)	30 (100%)	2.7/5 (SD 0.9)	4.0/5 (SD 0.4)

* other consists of dietician, midwife, and physician-clinical psychologist. ** The pre-survey consisted of two questions while the post-survey consisted of nine questions (all using a scale of 1–5). See Appendix A and Appendix B for the full content of the pre-survey and post-survey.

**Table 4:** Comparison of pre-survey and one-year post-survey utilization of palliative care.

Question	Responses (N)	Average Score (Standard Deviation)	Median Score [Interquartile Range]
Pre-Survey	32	2.9 (1.2)	2.5 [2.0, 4.0]
One-Year Post-Survey	9	4.9 (0.3)	5.0 [5.0, 5.0]

Responses to pre-survey Q3 and one-year post-survey Q7, both of which prompt the respondents to the frequency of which they utilize principles of palliative care medicine in their medical practice. See Appendix A and Appendix B, and D for the full content of the pre-survey and post-survey questions.

## Data Availability

Data is unavailable to protect the privacy of participating health care providers, but is available upon request for the purpose of peer review.

## Appendix A

### Pre-Test Survey

- What is your current education level?
  ○ Attending physician/specialist
  ○ Resident physician
  ○ Medical officer
  ○ Medical student
  ○ Nurse
  ○ First responder
  ○ Psychologist
  ○ Other (please list) ___________________________
- 1. Why did you enroll in this course?
  ○ Educational requirement
  ○ Preparation for exams
  ○ Want to learn new skills
  ○ Want to improve existing skills
  ○ Other (please list) ____________________________
- 2. Compared to your peers, how would you rate your knowledge of palliative medicine and/or palliative techniques?
  ○ I do not have significant baseline knowledge of palliative medicine
  ○ Below average
  ○ Average
  ○ Above average
  ○ Expert
- 3. In a given month, how often do you believe you utilize principles of palliative medicine in the care of your patients?
  ○ Never
  ○ Once a month
  ○ Once a week
  ○ Frequently
  ○ Multiple times per work day
- Please enter your email for follow-up purposes ______________________________________
- Please enter your WhatsApp for follow-up purposes ___________________________________

## Appendix B

### Post-Test Survey

- What is your current educational level?
  ○ Attending physician/specialist
  ○ Resident physician
  ○ Medical officer
  ○ Medical student
  ○ Nurse
  ○ First responder
  ○ Psychologist
  ○ Other (please list) ___________________________
- 1. The course content was well-organized
  ○ Strongly disagree
  ○ Somewhat disagree
  ○ Neither agree nor disagree
  ○ Somewhat agree
  ○ Strongly agree
- 2. The lectures presented were effective in helping me understand the topics presented
  ○ Strongly disagree
  ○ Somewhat disagree
  ○ Neither agree nor disagree
  ○ Somewhat agree
  ○ Strongly agree
- 3. The small group sessions enhanced the material presented in lectures
  ○ Strongly disagree
  ○ Somewhat disagree
  ○ Neither agree nor disagree
  ○ Somewhat agree
  ○ Strongly agree
- 4. How did the content presented rank in complexity given your educational background?
  ○ Too complicated and too difficult to understand most concepts
  ○ Somewhat complicated. I was able to understand the main points only
  ○ Desired level of complexity
  ○ Simple. However, I still found the information useful
  ○ Too simple. I would prefer a higher level of complexity
- 5. The instructors were effective
  ○ Strongly disagree
  ○ Somewhat disagree
  ○ Neither agree nor disagree
  ○ Somewhat agree
  ○ Strongly agree
- 6. The length of the lectures were
  ○ Much too short
  ○ Too short
  ○ Just the right length
  ○ Too long
- 7. Rate the likelihood that you are going to use the concepts learned in your daily practice/work day
  ○ Not likely at all
  ○ Somewhat likely
  ○ Very likely
  ○ Plan to use the concepts learned as frequently as possible
- 8. How has your confidence in TBI management changed after taking this course?
  ○ Worse than prior
  ○ Similar to prior
  ○ Improved somewhat
  ○ Significantly improved
- 9. How has your knowledge of palliative medicine changed after taking this course?
  ○ Worse than prior
  ○ Similar to prior
  ○ Improved somewhat
  ○ Significantly improved
- Please enter any other comments below

## Appendix C

### Pre- and Post-Test Questions and Answers

1. Which of the following is/are core principles of palliative care that should considered for every eligible patient?
  - Goals of care discussions
  - Symptom management
  - Hospice referral
  - **A + B**
  - All of the above
2. Which of the following patients would benefit MOST from palliative care medicine or a palliative care consult?
  - An 81-year-old female with a history of end-stage breast cancer, currently residing at a hospice facility
  - A 74-year-old male who is otherwise healthy, who has previously filled out a Do-Not-Resuscitate order
  - **A 19-year-old female athlete, with a history of anxiety, admitted to a hospital after a motor vehicle accident, with bilateral traumatic below knee amputations**
  - A 35-year-old male, admitted to the ICU after a tree fell on his head, with a non-survivable brain injury, currently on mechanical ventilation, with plans to undergo formal brain death testing
3. Which of the following suggests that shared decision-making was successful?
  - Family members and patients express an understanding of the relevant medical literature
  - **A decision was made that is mutually acceptable to the patient, family, and clinicians**
  - Physicians discussed relevant medical literature with the patient and family members
  - Family members experience grief regarding decisions to move away from curative care
4. Each of the following statements is true, except
  - Palliative medicine seeks to relieve suffering of physical, psychosocial, and spiritual origin
  - When facilitating goals of care conversations with a patient and his/her/their family, offering a treatment recommendation is considered appropriate
  - **Early incorporation of palliative medicine in critically ill patients results in increased in-hospital and 30-day mortality**
  - Early incorporation of palliative medicine in critically ill patients results in an increase in hospice referrals
  - In addition to patient-centered care, palliative medicine enhances the experience of family members, caregivers, and health care professionals
5. A 33-year-old male patient with diffuse axonal injury is admitted to the ICU. On day 5, the patient develops sudden diaphoresis, with a BP of 240/150, temperature of 39.8 C despite a cooling blanket, diaphoresis, and decorticate posturing. Which of the following is considered a first line preventative treatment for this condition?
  - Haloperidol
  - Propofol
  - **Propranolol**
  - Lorazepam
  - Gabapentin
6. You are caring for an 82-year-old woman suffering from severe anoxic brain injury after a cardiac arrest. The family is considering a tracheostomy versus transition to comfort measures only. During a conversation with the family, the patient’s daughter and health care surrogate expresses that she is overwhelmed with the decision-making process. She asks you “if this were your mother, what would you do?” What is an appropriate way to respond to this question?
  - Do not respond—it is inappropriate to answer, because all patient’s wishes are different
  - Provide a response only if it is in line with the decision you expect the family to make
  - Cite neuroprognostication data, and make a recommendation based on the probabilities of a good outcome
  - **Make a recommendation based on what you understand to be the patient’s treatment preferences and goals of care**
  - Explain that the decision rests solely on the health care surrogate and family, and any information you provide would be considered persuasion
7. Which of the following can improve delirium in a post-TBI patient?
  - Starting Lorazepam at night for sleep
  - Avoiding enteral nutrition due to high risk of aspiration
  - Avoiding narcotics for pain control
  - **Minimizing night-time stimulation, blood draws, and neurologic checks**
8. Each of the following are strategies care providers can employ to express empathy, except
  - Naming an emotion that a family member is expressing, and acknowledging it as acceptable
  - Statements that express that you understand what the patient or family member is saying
  - Respecting the contributions of family members, even if these contributions are unreasonable or factually incorrect
  - **Providing an anecdote about your personal experience that is similar to the patient’s experience, and what you did to overcome the challenge**
  - Asking questions to explore a patient or family member’s statement further
9. You are caring for a 45-year-old female with end-stage ovarian cancer. She suffers from chronic pain, and nausea related to her chemotherapy. During your evaluation, you find the patient to be febrile, and short of breath. She is considering hospice. Which of the following should be considered as pharmacologic intervention to improve comfort at the end of life?
  - Prednisone for chronic pain
  - Haloperidol for nausea
  - Inhaled albuterol for dyspnea
  - Oral morphine for pain
  - **All of the above**
10. Which of the following is true regarding elevated intracranial pressure in TBI?
  - In elevated ICP, the systemic blood pressure must be kept low to reduce cerebral blood flow which increases space for the brain to swell, based on the monro-kellie doctrine
  - A P2 > P1 on the intracranial monitor wave form is normal
  - **Patients with ICP > 20 for total duration > 40 min tend to have worse outcomes**
  - Early bifrontal craniotomy has been proven to reduce mortality, and improve long-term functional outcomes in elevated ICP secondary to TBI
11. You are caring for an 81-year-old patient who has suffered a catastrophic stroke which has permanently compromised her swallowing function. The family wishes to continue with aggressive curative care, but has refused a surgical enteric feeding tube because they believe the patient will recover her swallowing function. They reference a family friend that was on a ventilator for several weeks after an abdominal surgery who was able to swallow safely after tracheostomy. Which of the following is the most effective way to address this issue, without alienating the family?
  - Recovering the swallowing function is medically impossible and unrealistic. If we don’t place a feeding tube now, there is a high risk that she will not meet her nutritional needs
  - We can wait to see if she recovers her swallowing function and readdress the issue in the next few weeks
  - **I wish it were possible for her to rapidly recover her swallowing function; however, I worry that the stroke has damaged the parts of her brain that allow her to safely meet her nutrition needs without a feeding tube**
  - If the patient tries to eat on her own, she will be at risk of choking to death or developing aspiration pneumonia, which could be potentially life threatening and compromise all the hard work we’ve put into her care
12. A 32-year-old female has been admitted to the ICU after a fall, sustaining a TBI. Initial CT brain scan demonstrated bilateral subarachnoid hemorrhage and a 2 cm Rt frontal hemorrhagic contusion, with slightly decreased gray-white differential. She has been admitted for a week, and has had numerous ICP crises requiring sedation with propofol, osmotherapy, and a paralytic infusion. Off sedation and paralytic for 24 h, the patient has no spontaneous respirations, no motor response to pain, and fixed dilated pupils. All most recent blood work is within normal limits. Which of the following is true regarding this patient?
  - The patient is likely brain dead, and should undergo immediate formal brain death testing
  - **A repeat CT brain scan should be obtained to evaluate for catastrophic brain injury**
  - The patient’s physical exam is not consistent with brain death
  - The patient should be observed for a longer time to allow for propofol to be entirely metabolized from her system
13. What is secondary brain injury?
  - Injury caused after direct blunt impact by the brain recoiling against the skull, causing injury on the opposite side of the brain
  - Brain injury due to surgical/procedural related trauma
  - Injury due to a second traumatic event
  - **Injury due to acute post-TBI-related ischemic, metabolic, and inflammatory sequelae**
14. Which best describes the pathophysiology of paroxysmal sympathetic hyperactivity syndrome?
  - Damage to the stellate ganglion nucleus leads to release of catecholamines and hemodynamic instability
  - **Loss of inhibition of the periaqueductal gray nucleus leads to exaggerated sympathetic responses to both noxious and normal stimuli**
  - Brain herniation leads to compression of the medulla, leading to irregular breathing patterns, hypertension, and bradycardia
  - Severe brain injury causes secondary coronary ischemia leading to hypertension, tachycardia, and heart failure
15. Which of the following is considered a tier 2 therapy for the management of an ICP crisis?
  - **Infusion of a paralytic medication**
  - Osmotherapy with hypertonic saline
  - Therapeutic hypothermia
  - Elevating the head of the bed 30–45 degrees
16. Which of the following is false regarding treating elevated ICP?
  - Starting an infusion of hypertonic saline empirically to target a supratherapeutic sodium goal does not improve long-term functional outcomes
  - Decompressive craniotomy improves all cause in-hospital mortality in severe TBI patients
  - Barbiturate coma is a tier 3 therapy and should only be reserved for refractory ICP crises
  - **Hyperventilation to a PaCO_2_ goal 30–35 should be instituted in all TBI patients on mechanical ventilation**
17. A 50-year-old male presents after a TBI and has a small area of hemorrhage in the anterior pons. You are concerned about the patient having Locked-In syndrome. Which neurologic exam finding may confirm your suspicions?
  - Diffuse hyperreflexia
  - **The patient is able to look downward on command**
  - Spontaneous roving eye movements
  - No response to verbal or noxious stimuli
18. What is the first step in shared decision-making?
  - Comprehension of available literature and guidelines surrounding a decision
  - Assessment of your patient’s values and preferences
  - **Determining that a decision needs to be made**
  - Explaining all treatment options and the respective pros and cons
19. Which of the following is FALSE regarding neuroprognostication after TBI?
  - **Patients that remain comatose after two weeks are unlikely to have improvements in their neuro exams**
  - Age is a predictor of bad outcome in TBI
  - Lack of pupillary response at 72 h is one of the most reliable predictors of a poor outcome
  - Self-fulfilling prophecy bias occurs when a prediction of a patient’s outcome directly leads to the fulfillment of that outcome
20. A 20-year-old male presents with a TBI suffered 24 h ago, and is found to have diffuse axonal injury and multiple cerebral contusions. He has sluggishly reactive 4 mm pupils bilaterally, withdraws all extremities to pain, and does not open his eyes. During your evaluation, the patient has a generalized seizure. The patient’s father asks you if the patient is going to be able to walk again. What is the most appropriate response?
  - It is too early to tell, but after 72 h I will be able to answer questions about his neurologic outcome and likelihood of survival
  - Based on the patient’s neuro exam, if he survives this hospitalization, he will likely be in a persistent vegetative state permanently
  - **I do not have enough information to portend a prognosis; however, over the course of several months and with intensive physical therapy his condition may improve**
  - The patient’s pupillary exam strongly predicts a poor outcome
21. Which of the following TBI subtypes matches the appropriate mechanism and clinical pearl?
  - **Cerebral venous thrombosis—often associated with local skull fracture—can lead to secondary intracranial hemorrhage**
  - Epidural hematoma—etiology is rupture of bridging veins—lucid interval followed by rapid deterioration
  - Subdural hematoma—damage to middle meningeal artery branch off external carotid artery—hourglass appearance on imaging
  - Diffuse axonal injury—mechanism is axonal shear—vasospasm is common
22. Which of the following is a true statement?
  - Shared decision-making is not recommended when family members lack the medical literacy to meaningfully participate
  - A dialogue is a conversation in which a physician explains a diagnosis, and offers treatment options
  - A principle of shared decision-making is arriving to a clinical decision that primarily the patient and his/her family finds acceptable
  - **A clinician’s adequate understanding of the related medical literature and evidence-based medicine is necessary to successfully employ shared decision-making**
23. What is the proper way to test corneal reflex when undergoing brain death testing?
  - Light pressure to the lateral conjunctiva with a cotton swab
  - A saline flush dripped on the open conjunctiva
  - Light pressure directly over the pupil with gauze
  - **Moderate pressure to the medial conjunctiva near the iris**
24. A 19-year-old male presents after a MVA, with a severe TBI. He is intubated prior to arrival to the hospital. A CT brain scan demonstrates large Rt subdural hematoma, with multiple left-sided subarachnoid hemorrhages. He undergoes an Rt decompressive hemicraniectomy, and arrives to the ICU stable. After shared decision-making, the family has decided to pursue aggressive curative care. When should you meet again with the family to rediscuss goals of care, and to ensure the patient’s currently described goals of care are adequately being met?
  - **Within 24 h**
  - Within 1 week
  - Only if the patient has a significant clinical change
  - Once it is time to consider tracheostomy
  - Only if it becomes clear the patient will not survive without a major disability
25. Which of the following is true regarding the principle of the Lassen curve of cerebral autoregulation?
  - Every TBI patient has a static individual range of safe blood pressure, where changes in SBP do not significantly affect cerebral blood flow
  - A history of prolonged hypertension may shift a patient’s autoregulation curve leftward
  - A slope of close to 0 signifies severe compromise of a patient’s cerebral autoregulation curve
  - **In severe TBI with cerebral edema and blood-brain-barrier compromise, even small changes in blood pressure can dramatically influence cerebral blood flow**

## Appendix D

### One-Year Post-Course Survey Questions

1. What is your current education level?
  - Attending physician/specialist
  - Resident physician
  - Medical officer
  - Medical student
  - Nurse
  - First responder
  - Psychologist
  - Other (please list) ___________________________

For the following questions, indicated on a scale of 1–5

2. How has your confidence in TBI management changed after taking this course?
  1. Worse/more confused than before
  2. Not changed
  3. Improved but not notably
  4. Somewhat improved
  5. Greatly improved
3. How has your knowledge of palliative medicine changed since taking this course?
  1. Worse/more confused than before
  2. Not changed
  3. Improved but not notably
  4. Somewhat improved
  5. Greatly improved
4. Since taking this course, how have your views changed on TBI management?
  1. Not at all
  2. Between not at all and somewhat
  3. Somewhat changed
  4. Between somewhat and greatly changed
  5. Greatly changed
5. How often do you utilize concepts learned from the TBI portion of the course?
  1. Never
  2. Between never and sometimes
  3. Sometimes
  4. Between sometimes and frequently
  5. Frequently
6. Since taking this course, how have your views changed on palliative medicine?
  1. Not at all
  2. Between not at all and somewhat
  3. Somewhat changed
  4. Between somewhat and greatly changed
  5. Greatly changed
7. How often do you utilize concepts learned from the palliative care section of the course?
  1. Never
  2. Between never and sometimes
  3. Sometimes
  4. Between sometimes and frequently
  5. Frequently—every day
8. Since taking the course, how has your approach to breaking bad news changed?
  1. Not at all
  2. Between not at all and somewhat
  3. Somewhat changed
  4. Between somewhat and greatly changed
  5. Greatly changed
9. How often do you use a strategy learned in this course when discussing bad news with patients or families?
  1. I don’t use a strategy learned in this course
  2. I want to, but cannot remember how
  3. Rarely
  4. Sometimes
  5. Often
10. How often do you utilize shared decision-making in your discussions with patients?
  1. Never
  2. Between never and sometimes
  3. Sometimes
  4. Between sometimes and frequently
  5. Frequently—every day
11. Since taking the course, how frequently do you take into account patient/family treatment preferences before coming to a decision or making a recommendation on treatment?
  1. Never
  2. Between never and sometimes
  3. Sometimes
  4. Between sometimes and frequently
  5. Frequently
12. Since taking the course, have you had a patient that required withdrawal of life sustaining treatment?
  1. Yes
  2. No
13. If yes, how much did you find the course material relevant?
  1. Not relevant
  2. I found it relevant, but did not remember how to respond
  3. Somewhat relevant
  4. Very relevant
  5. Extremely important/glad I took the course
14. If yes, how did the course material change the way you approached these cases?
  1. Not at all
  2. Between not at all and somewhat influential
  3. Somewhat influential
  4. Between somewhat and highly influential
  5. Highly influential
15. Since taking the course, how often do you consider treating painful or distressing symptoms using palliative care principles?
  1. Never
  2. Between never and sometimes
  3. Sometimes
  4. Between sometimes and frequently
  5. Frequently
16. Having taken the course, knowing what you know now, would you take it again?
  1. Definitely not
  2. Unlikely
  3. Possibly
  4. Very likely
  5. Definitely
17. Would you recommend the course to your colleagues?
  1. Definitely not
  2. Unlikely
  3. Possibly
  4. Very likely
  5. Definitely
18. Overall, rate the influence the course has had on your career
  1. Negatively influential
  2. Not helpful
  3. Neutral
  4. Helpful
  5. Positively influential

## Appendix E

**Table A1:** One-year post-course survey responses.

Question		Total Cohort (n = 9)	Question Score Average
What is your current education level?	Attending physician/specialist	2 (22%)	-
	Medical officer	1 (11%)	
	Nurse	6 67%)	
How has your confidence in TBI management changed after taking this course?	Worse		4.6
	Not changed		
	Improved, not notably	1 (11%)	
	Somewhat improved	1 (11%)	
	Greatly improved	6 (67%)	
	No response	1 (11%)	
How has your knowledge of palliative care changed since taking this course?	Worse		4.9
	Not changed		
	Improved, not notably		
	Somewhat improved	1 (11%)	
	Greatly improved	8 (89%)	
Since taking this course, how have your views changed on TBI management?	Not at all		4.2
	Between not at all and somewhat	1 (11%)	
	Somewhat changed		
	Between somewhat and greatly	4 (44%)	
	Greatly changed	4 (44%)	
How often do you utilize concepts learned from the TBI portion of the course?	Never	2 (22%)	3.2
	Sometimes	4 (44%)	
	Frequently	3 (33%)	
Since taking this course, how have your views changed on palliative medicine?	Not at all		5.0
	Somewhat changed		
	Greatly changed	9 (100%)	
How often do you utilize concepts learned from the palliative care section of the course?	Never		4.7
	Sometimes	1 (11%)	
	Between sometimes and frequently	1 (11%)	
	Frequently	7 (78%)	
Since taking the course, how has your approach to breaking bad news changed?	Not at all		4.9
	Somewhat changed		
	Between somewhat and greatly	1 (11%)	
	Greatly changed	8 (89%)	
How often do you use a strategy learned in this course when discussing bad news with patients or families?	I don’t		5.0
	I want to, but can’t remember		
	Rarely		
	Sometimes		
	Often	9 (100%)	
How often do you utilize shared decision-making in your discussion with patients?	Never		3.8
	Sometimes	5 (56%)	
	Between sometimes and frequently	1 (11%)	
	Frequently	3 (33%)	
Since taking the course, how frequently do you take into account patient/family treatment preferences before coming to a decision or making a recommendation on treatment?	Never		3.2
	Sometimes	8 (89%)	
	Frequently	1 (11%)	
Since taking the course, have you had a patient that required withdrawal of life sustaining treatment?	Yes	3 (33%)	No
	No	6 (67%)	
If yes (n = 3), how much did you find the course material relevant?	Not relevant		4.7
	Relevant, but could not remember		
	Somewhat		
	Very relevant	1 (33%)	
	Extremely important	2 (67%)	
If yes (n = 3), how did the course material change the way you approached these cases?	Not at all	-	4.0
	Somewhat influential	1 (33%)	
	Between somewhat and highly	1 (33%)	
	Highly influential	1 (33%)	
Since taking the course, how often do you consider treating painful or distressing symptoms using palliative care principles?	Never		4.3
	Sometimes	2 (22%)	
	Between sometimes and frequently	2 (22%)	
	Frequently	5 (56%)	
Having taken the course, knowing what you know now, would you take it again?	Definitely not		4.8
	Unlikely		
	Possibly		
	Very likely	2 (22%)	
	Definitely	7 (78%)	
Would you recommend the course to your colleagues?	Definitely not		4.9
	Unlikely		
	Possibly		
	Very likely	1 (11%)	
	Definitely	8 (89%)	
Overall, rate the influence the course has had on your career	Negatively influential		4.9
	Not helpful		
	Neutral		
	Helpful	1 (11%)	
	Positively influential	8 (89%)	
